# A versatile liquid-jet setup for the European XFEL^1^

**DOI:** 10.1107/S1600577519000894

**Published:** 2019-02-22

**Authors:** J. Schulz, J. Bielecki, R. B. Doak, K. Dörner, R. Graceffa, R. L. Shoeman, M. Sikorski, P. Thute, D. Westphal, A. P. Mancuso

**Affiliations:** a European XFEL, Holzkoppel 4, Schenefeld 22869, Germany; b Max-Planck-Institut für Medizinische Forschung, Jahnstrasse 29, D-69120 Heidelberg, Germany; c Department of Cell and Molecular Biology (ICM), Uppsala University, Husargatan 3, Uppsala 75124, Sweden; d Department of Chemistry and Physics, La Trobe Institute for Molecular Science, La Trobe University, Melbourne, Victoria 3086, Australia

**Keywords:** FEL physics, instrumentation, liquid jets, sample delivery

## Abstract

The SPB/SFX instrument of the European XFEL provides unique possibilities for high-throughput serial femtosecond crystallography. This publication presents the liquid-jet sample delivery setup of this instrument. The setup is compatible with state-of-the-art gas dynamic virtual nozzle systems as well as high-viscosity extruders and provides space and flexibility for other liquid injection devices and future upgrades. The liquid jets are confined in a differentially pumped catcher assembly and can be replaced within a couple of minutes through a load-lock. A two-microscope imaging system allows visual control of the jets from two perspectives.

## Abstract 2

A versatile liquid-jet injection system for serial femtosecond crystallography at the SPB/SFX instrument of the European XFEL is presented. The system comprises a load-lock nozzle rod changer, chamber-in-chamber differential pumping, liquid handling systems and two microscopes for sample imaging and diagnostics.

## 1. Motivation   

One of the goals of the Single Particles, Clusters, and Biology and Serial Femtosecond Crystallography (SPB/SFX) instrument (Mancuso *et al.*, 2019 ▸) at the European XFEL is enabling users from around the world to perform protein crystallography experiments on micrometre- and sub-micrometre-sized crystals suspended in liquid buffer solutions. The setup of such a serial femtosecond crystallography (SFX) experiment consists of a liquid jet a few micrometres in diameter being hit by X-ray pulses. Bragg scattering from the protein crystals is collected shot-by-shot in a large angle to allow optimal spatial resolution. The exact geometry of the liquid jets depends on details of the planned experiment and the sample. Being a user facility, the European XFEL provides a basis for a vast set of future liquid-jet-based experiments.

Important liquid jet injection devices are gas dynamic virtual nozzles (GDVNs) and high-viscosity extruders. GDVNs use a gas stream to further compress a 50–100 µm jet from a capillary. These jets reach diameters down to a few micrometres and speeds in the range 20–100 m s^−1^. Membrane proteins can often be crystallized in the lipid cubic phase; here, high-viscosity extruders are needed to produce a constant stream of a high-viscosity fluid. One strength of free-electron lasers (FELs) is the short bunch lengths that allow high time resolution. For time-resolved measurements, laser pump beams or fast-mixing devices need to be integrated into the experimental setup.

Liquid jets are potential sample environments for other instruments at the European XFEL, such as Material Imaging and Dynamics (MID) or Small Quantum Systems (SQS). The liquid-jet sample environment is designed to allow easy transfer to other instruments. The modular design of the setup allows easy changing of the setup for new experimental demands as well as modifications for use in other instruments.

## 2. Design goals and requirements   

X-ray FELs provide femtosecond short X-ray pulses with peak power densities nine orders of magnitude higher than conventional synchrotron radiation sources. A unique feature of the European XFEL is the bunch structure: 600 µs-long pulse trains are generated with a 10 Hz repetition rate. Once the machine has achieved full design specifications, up to 2700 X-ray pulses with a repetition rate of 4.5 MHz can be created within each pulse train. This structure provides 27 000 pulses per second. At full repetition rate, the 220 ns between two adjacent pulses in a train poses a challenge for sample replacement, which is necessary due to single-shot sample destruction by the focused X-ray FEL beam.

The adaptive gain integrating pixel detector (Allahgholi *et al.*, 2016 ▸; Mezza *et al.*, 2016 ▸) used at the SPB/SFX instrument can record images with 4.5 MHz within a pulse train and transfer them into the data acquisition system within the 99 ms between pulse trains. This active pixel detector has a storage capacity of 352 images, limiting the effective maximum image repetition rate to 3520 pulses per second. Even more pulses can be used by vetoing the detector when no crystal has been hit. The design goal of the European XFEL is to share the 2700 pulses of the pulse trains between the three X-ray undulators. Therefore, the sample delivery and detector system should be designed to accept the delivered pulses in as high a repetition rate as achievable so that the remaining pulses can be directed to other instruments.

The SPB/SFX instrument will ultimately focus the X-ray pulses with a choice of two Kirkpatrick–Baez mirror systems. The micrometre-focusing system is designed to deliver a beam diameter of the order of 1 µm. With the nano-focusing system, the beam spot diameter can be reduced by another order of magnitude to about 100 nm (Bean *et al.*, 2016 ▸).

The sample chamber of the SPB/SFX instrument is kept under high vacuum to minimize background radiation and avoid arcing in the detector electronics. In order to keep reasonable vacuum conditions in the main sample chamber, the liquid jets need to be driven in a separately pumped shroud. To ensure effective differential pumping, the shroud should have only a small entrance hole for the X-rays and an exit cone for the scattering signal.

For alignment and visual feedback of the jetting process, at least two microscopes have to be available to inspect the interaction from two different angles, ideally one microscope in line with the X-ray beam and the other orthogonal from the side. This requires view ports in the shroud for microscope inspection and illumination. Additional view ports are needed for in- and out-coupling of a femtosecond pump–probe laser.

Alignment of the catcher shroud with two holes requires five degrees of freedom: centring the interaction point requires three linear displacements, aligning the two holes with the beam requires two rotations. With X-ray holes of the order of 1–3 mm, the precision of this alignment does not need to be better than 10 µm. Because of the large vacuum chamber, the actuators for this alignment system can only be placed in a vacuum. The group investigated two options: (i) a stack of three linear stages, a rotation stage and a goniometer stage with vacuum-compatible stepper motors and (ii) an in-vacuum hexapod. Since the first option – a five-axes manipulator stack – would be far too bulky even for the relatively large chamber, a three-axes manipulator has been discussed as well. In this latter constellation, alignment of the holes would be ensured by machining precision.

Considering the two possible designs, the team decided to implement a customized compact commercial hexapod to mount the catcher system. This system is even more compact than the three-axes linear stage assembly and provides a much higher resolution than necessary. It can therefore also serve as a basis for precise fixed-target positioning.

## 3. Nozzle rod and catcher system   

### 3.1. Liquid-free-jet sample injection system   

A rod-based injector system was constructed for the SPB/SFX instrument of the European XFEL. Design and fabrication were carried out at the Max Planck Institute for Medical Research in Heidelberg, Germany. The injector system is similar in many regards to previous systems designed by R. B. Doak (Weierstall *et al.*, 2012 ▸) and employs the same GDVNs that made X-ray FEL studies of biological species possible (DePonte *et al.*, 2008 ▸; Doak & Shoeman, 2018 ▸). Also supplied was an ‘anti-settling’ device similar to that previously described (Lomb *et al.*, 2012 ▸). All custom parts of the injector system were fabricated at the Max Planck Institute, as was the anti-settling device in its entirety.

**Figure 1:**
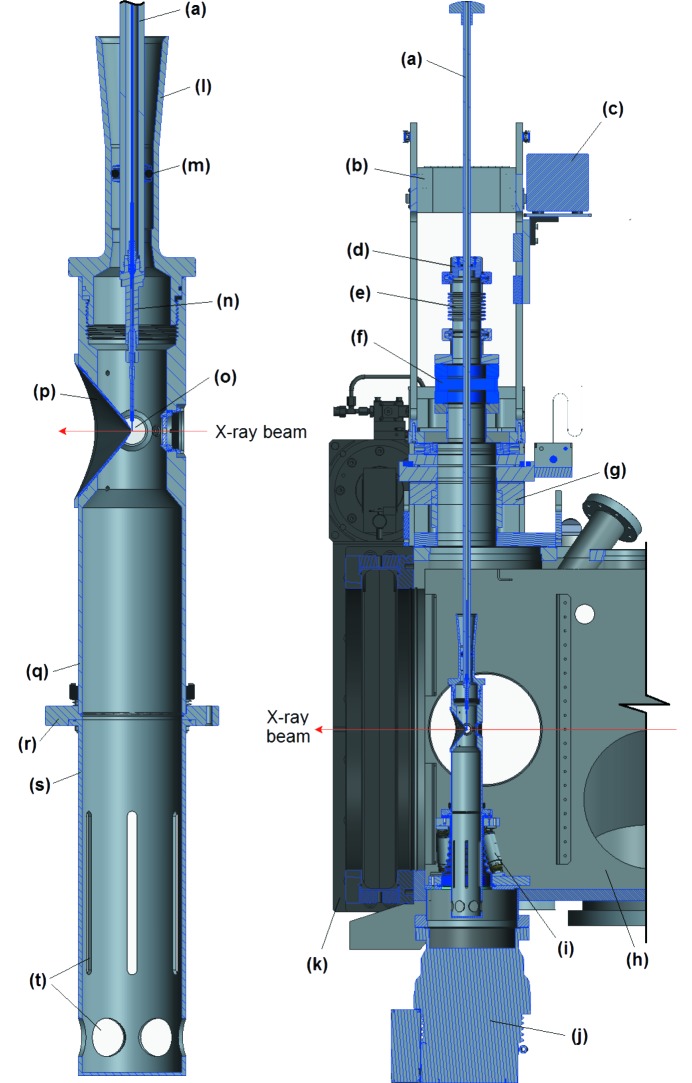
Rod-based GDVN injection system designed and constructed for use in the SPB/SFX chamber. Detailed descriptions of the components labelled (a) to (t) are found in Section 3.1.

Two cross-sectional CAD drawings of the injector system are shown in Fig. 1 ▸. The components form a stack extending from top to bottom across the SPB/SFX scattering chamber. The entire stack is seen in the right-hand image of Fig. 1 ▸. An enlarged view of the lower end of the stack is presented on the left. The cross-sectional cuts of Fig. 1 ▸ are in the vertical plane containing the X-ray beam, the path of which is indicated in both images. The various components of the injector system are labelled.

(*a*) The injector rod is a stainless steel tube of 12.70 mm (outer diameter, OD) × 6.60 mm (inner diameter, ID) and 1215 mm in length. The rod has an M9×1 internal thread on its bottom end for attachment of the nozzle section and a knob for gripping and rotating the rod on its upper end. Capillaries to supply GDVN gas and sample solution to the nozzle pass from the nozzle section up through the rod to emerge from the top end.

(*b*) A rigid superstructure framework mounts a guide jig to guide the rod during its insertion into the scattering chamber. Once the rod has been inserted, the guide block flips backward on a hinge to fully disengage from the rod, leaving the upper end of the rod free to move.

(*c*) A high-performance liquid chromatography (HPLC) valve is also mounted on the superstructure framework. The sample solution capillary from the nozzle connects to one of these ports, allowing it to be supplied either with sample solution from a temperature-controlled reservoir on the anti-settling device or with ultrapure water.

(*d*) The nozzle rod slides through an O-ring seal built into a KF50 ‘top-hat’ that forms the top of the injector vacuum stack. This O-ring seals the ultrahigh vacuum environment inside the chamber from atmospheric ambient conditions. A knurled screw in the top-hat can clamp the rod relative to the top-hat.

(*e*) A KF50 bellows section below the top-hat provides the flexure needed to accommodate motion of the upper end of the rod. Immediately below this bellows is a CF63 to KF50 vacuum flange adaptor.

(*f*) The CF63 to KF50 adaptor mounts on top of a pneumatically operated commercial vacuum gate valve, forming a load-lock section above the gate valve. With the gate valve closed, the nozzle rod is inserted into the bellows/adaptor section and the KF50 clamp is placed in position and tightened to seal the plenum above the gate valve. This region can then be evacuated to good fore-vacuum by means of a mechanical pump that connects to the plenum through the lower flange of the adaptor. The gate valve is then opened and the rod is inserted into the scattering chamber.

(*g*) The gate valve mounts atop a specially built commercial *XYZ* manipulator. By this means, the upper end of the rod can be moved horizontally, either parallel or perpendicular to the X-ray beam as well as vertically perpendicular to the beam.

(*h*) The *XYZ* manipulator is mounted on the top of SPB/SFX scattering chamber.

(*i*) The lower section of the injector stack mounts on a hexapod manipulator that is bolted to the bottom of the chamber. A flexible, contiguous vacuum envelope leads down through the centre of the hexapod and seals the bottom of the chamber around a tubulation that leads through the bottom of the chamber.

(*j*) A turbomolecular pump, situated below the chamber, is bolted to the other end of this tubulation. Together, these lower components of the injector shroud comprise a differential pumping plenum to limit the pressure rise in the scattering chamber even when considerable GDVN gas is flowing through the nozzle section.

(*k*) A 500 mm gate valve on the detector end of the scattering chamber is opened for measurements and admits the front end of the detector for measurements at very close approach to the free jet.

(*l*) Topmost in the lower section of the injector stack is a funnel-shaped receptor that guides insertion of the nozzle rod. The lower injection section is completely detached from the upper section when the nozzle is not inserted. Once inserted, the rod links the two, but, by means of the flexible O-ring seals and the bellows section at the top of the stack, it is a very flexible connection.

(*m*) The funnel tapers from 31 mm diameter at its mouth down to a 22.00 mm internal bore. An O-ring fixed to the rod slides into this bore, sealing the differential pumping plenum below the funnel from the scattering chamber outside.

(*n*) A nozzle holder (Weierstall, 2014 ▸; Doak & Shoeman, 2019 ▸) screws into the end of the nozzle rod.

(*o*) The GDVN nozzle shown is comprised of a gas sheath of stainless steel tubing with a borosilicate insert. Within the insert, a coned fused silica capillary is situated (Doak *et al.*, 2019 ▸).

(*p*) A replaceable re-entrant cone is bolted to the wall of the injector shroud. This provides a free solid angle of 100° for the diffracted X-rays, though does not overly compromise the differential pumping.

(*q*) The injector shroud opens up to an inner diameter of 52 mm below the nozzle, providing as much vacuum conductance as possible without interfering with the hexapod legs.

(*r*) The bottom end of the injector shroud is flanged for bolting onto the upper plate of the hexapod. This connection also clamps into place the ‘catcher’ can that sits below the injector shroud.

(*s*) The catcher can collects efflux from the nozzle. It lifts upwards out of its recess once the injector shroud above it has been removed.

(*t*) Slots and holes through the lower sidewall of the can provide about 400 l s^−1^ of vacuum conductance for pumping of the can by the turbomolecular pump.

### 3.2. Options for larger nozzle devices   

As a user facility, European XFEL aims to enable users to bring in their own liquid sample injection devices. To overcome the space constraints of the half-inch nozzle rod system, we adapted the liquid-jet injector system at the SPB/SFX instrument to permit the in-vacuum use of devices with up to 28 mm OD. This will allow the use of new devices with mixing options, additional modules and control.

**Figure 2:**
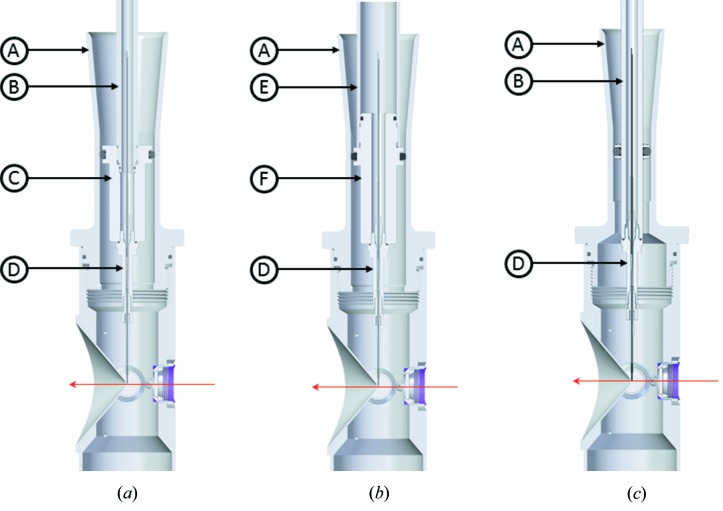
(*a*) Setup of the liquid sample delivery combining the large catcher top-hat (A) and the half-inch OD nozzle rod (B). The ID of the engaging region of the top-hat catcher (A) is 32 mm, allowing insertion of the adapter nut (C) and device (D) assembly. A 25 mm × 4 mm Viton O-ring engages the rod to the catcher and separates the differential pumping shroud from the rest of the chamber. The nut connects with the rod via M12 internal threads. (*b*) Setup for the 25 mm OD nozzle rod (E). The GDVN (D) is screwed to the rod via adapter nut (F) with M9 internal threads. The adapter is fastened to the rod with M23 external threads. The magenta arrow represents the X-ray direction. (*c*) The half-inch nozzle rod and catcher assembly for comparison.

To adapt larger devices, the standard catcher top-hat is replaced with a catcher top-hat of a 32 mm ID. Two versions of the setup are available. In the first version, an adapter nut is connected to the half-inch OD nozzle rod by an M12 internal thread, allowing for a device up to 28 mm OD to be placed inside the catcher [Fig. 2 ▸(*a*)]. The second version has a 25 mm OD nozzle rod [Fig. 2 ▸(*b*)] with a modified matching top O-ring rod-seal and guide/clamp of the superstructure. This setup can host larger sample feeding arrangements owing to its bigger inner diameter.

The 25 mm OD nozzle rod has the caveat of much larger vacuum forces pulling it into the chamber. We calculated the vacuum forces to be equivalent to about 1.6 kg for the half-inch rod and 6.5 kg for the large rod. The simple clamping screws of the top O-ring rod seal overcome the vacuum forces, keeping the nozzle rod from being pulled in. The stronger vacuum forces and higher friction of the large rod, however, require a greater strength of the operator during manual nozzle exchange.

#### 3.2.1. Example: integration of a microfluidic jetting device   

**Figure 3:**
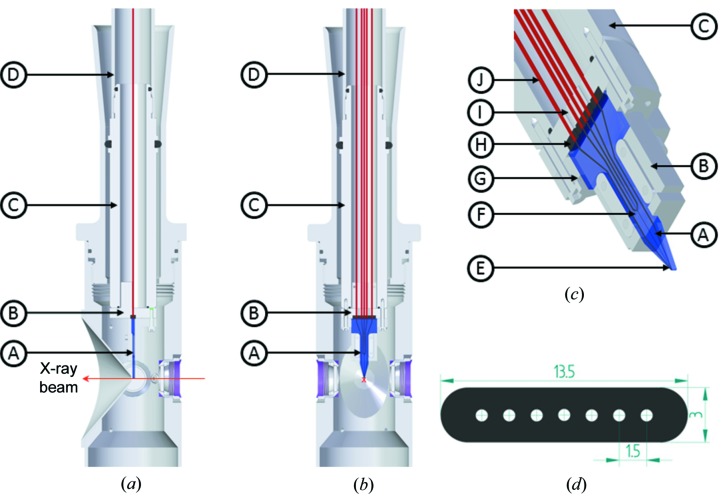
(*a*) Sectional side and (*b*) along the beam views of the microfluidics integrated into the SPB/SFX instrument. The magenta arrow represents the X-ray direction. The microfluidic device (A) and inline connector (B) are attached via a connector nut (C) fitting on the 25 mm OD nozzle rod (D) through M23 external threads. (*c*) Detailed view of microfluidic device and inline connector. The capillaries (J) are pushed into a seven-way gasket (H) and clamped by bolting the microfluidic device holder (G) with the connector for the capillaries (I) pressed against it. The microfluidic device has a mixing module (F) and a nozzle module (E) integrated. (*d*) Sectional view of the gasket shows dimensions defining the constraints for design of the microfluidic device.

Fig.3 ▸ shows an implementation of a sample-injection device utilizing the 25 mm OD nozzle rod. This device is a glass microfluidic device combining a mixing module with a GDVN jet in a monolithic piece.

The larger rod cross-section is used here to feed up to seven 1/32 in polyether ether ketone (PEEK) capillaries through the interface between the rod adapter and the microfluidic device. The capillaries terminate in a seven-way gasket with a push fit sealed assembly on the capillary side and a press fit seal at the inlet channels on the top surface of the microfluidic device. The assembly consists of a microfluidic holder and a connector for the capillaries. The holder, connector and injector rod are fitted together with the help of pins and screws, and sealed at various interfaces with O-rings. The microfluidics inlet side has a larger area to seal the inlets and tapers down towards the nozzle tip in order to cope with existing catcher space constraints.

The microfluidic device length is 35 mm. The width at the channel inputs is 15 mm, reduced to 0.3 mm at the nozzle tip. The thickness is 4 mm at the inlet, chamfered to 2.5 mm at the tip, with a distance of 0.5 mm between the channel centre and the microfluidic devices facing downstream of the X-ray interaction region. Furthermore, to avoid shielding of the scattering signal, the downstream surface is ground with an angle of 70°.

The device is fastened to the nozzle rod by a nut with M23 external threads. It is also possible to connect the device to the half-inch OD nozzle rod by using an adapter nut with M12 internal threads. This adapter nut then connects with the modified 32 mm ID catcher top head.

## 4. Sample preparation and delivery   

**Figure 4:**
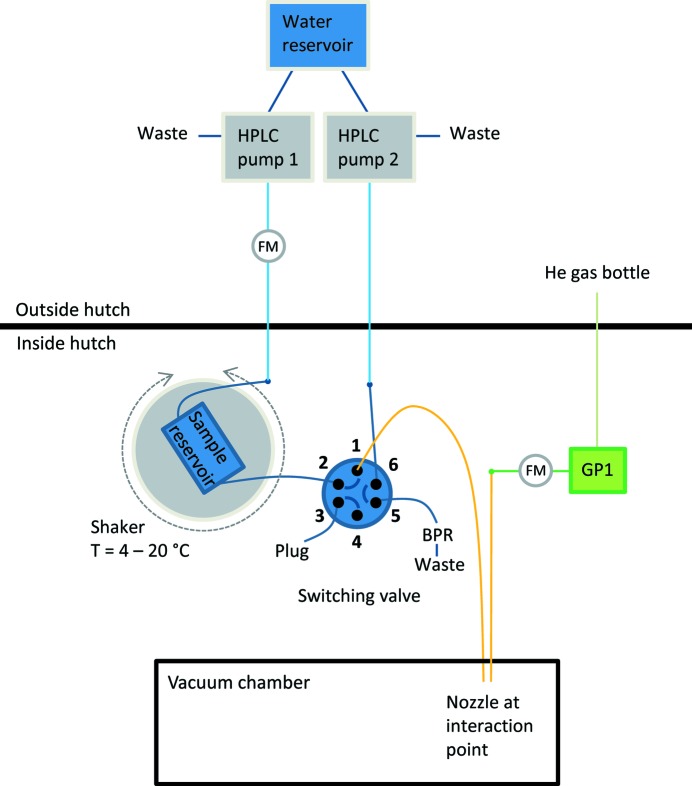
Scheme of a common injection setup at the SPB/SFX instrument. Liquid lines are shown in blue (dark blue: PEEK lines; light blue: stainless steel lines) and gas lines are green (dark green: PEEK lines; light green: stainless steel lines). Glass capillaries are shown in orange. Flow meters are indicated (FM). HPLC pumps are located outside of the experiment hutch. The connected waste lines enable quick pressure release in the liquid lines. The two-position, six-port switching valve connects either the sample reservoir (pressurized by pump 1) or the water line (pump 2) with the nozzle (connected to position 1). In the figure, the first case is shown. The back pressure regulator (BPR) ensures sufficient pressure in the water line when not connected to the nozzle. The sample reservoir is mounted on a rotating sample shaker and cooled down with a Peltier element. Helium pressure is provided by a pressure regulator (GP1).

At the SPB/SFX instrument, different pumping systems are available to enable sample delivery with GDVNs, the high-viscosity extrusion sample injector and the aerosol injector. Fig. 4 ▸ shows a sketch of a commonly used setup for liquid-jet injection: two HPLC pumps (LC-20AD, Shimadzu) equipped with a degassing unit (DGU-20 A5R, Shimadzu) provide degassed, deionized water. One pump is directly connected to the nozzle, the other pump pressurizes the sample reservoirs.

A two-position, six-port switching valve connects either the water line or the sample reservoir with the nozzle. The latter case is shown in Fig. 4 ▸. The sample reservoirs were designed and fabricated at the Max Planck Institute for Medical Research. They consist of an improved version of the high-pressure stainless steel syringe and plunger with dual Teflon seals, as described previously (Lomb *et al.*, 2012 ▸). The plunger separates the water side from the sample side. The HPLC pump pressurizes the water side, and, as a result, the plunger is pushing the sample out of the syringe to the nozzle.

Two different ways of operating the system are used. The pumps are located either in the experiment hutch on top of the sample chamber or in the laboratory next to the experiment hutch. In the first case, they are controlled remotely and directly connected to the PEEK pressure lines. In the latter, they can be operated locally, and the water is pumped via stainless steel lines to the PEEK lines at the experimental chamber.

As an alternative or in combination, syringe pumps can be used to provide a constant flow for sample injection. High-pressure modules (neMESYS 2600 N, Cetoni) and mid-pressure modules (neMESYS 1000, Cetoni) are available.

An additional option is a gas-driven system that pumps the liquid into the experimental chamber. Therefore, electronic pressure regulators (GP1, Poportion Air) are installed. These remotely controlled regulators are also used to provide the focusing gas for the GDVNs.

To enable the connection of several sample reservoirs or liquid lines in parallel, switching valves (Rheodyne MX Series II, IDEX Health & Science) are installed. Six-position, seven-port selector valves and two-position, six-port valves are available. Optionally, a two-position, six-port valve model (FCV-32AH, Shimadzu) can be used. The use of a two-position, six-port valve is depicted in Fig. 4 ▸. In the sample delivery position, the valve connects the output of the sample reservoir to the nozzle and the water line – through a back-pressure regulator (BPR) – to waste. When no sample is desired to flow through the nozzle, for example, during alignment, the other position of the valve closes the sample reservoir and guides the water into the nozzle. The six-position, seven-port selector valves are usually used in pairs to multiplex the pressure produced by one pump through up to five sample reservoirs and one bypass.

To monitor the liquid-flow rates, different flow meters covering a range from 100 nl min^−1^ to 3.3 ml min^−1^ water are available (mini CORI-FLOW and LIQUI-FLOW series, Bronkhorst; SLG and SLI series, Sensirion). Gas flow can be monitored in the range 2–100 mg min^−1^ helium (EL-FLOW Select series, Bronkhorst).

The entire setup is adjusted for each experiment and can be easily complemented with devices brought by users.

## 5. Sample environment imaging system   

Two microscopes are integrated into the liquid-jet sample environment. Both are based on Mitutoyo objectives of the series M Plan Apo with a 95 mm parfocal distance and 33.5–35 mm working distances. With magnifications of 2×, 5×, 7.5× and 10×, numerical apertures of 0.055–0.28 can be reached. For high resolution, an objective with 20× magnification and a 20 mm working distance can be inserted. This objective reaches a numerical aperture of 0.42, but is not compatible with the current catcher design.

**Figure 5:**
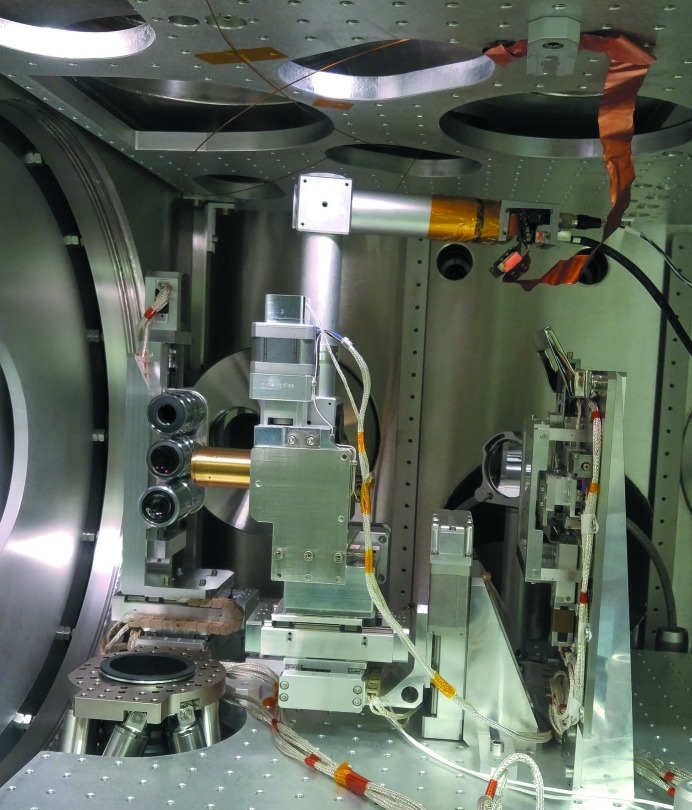
Image of the sample environment imaging system. In the centre, the Z-shaped inline microscope with its five-axis stepper motor-driven alignment system is visible. The catcher system has been removed to give the sight free to the three-sided view objectives in the background to the left side of the inline microscope. Below the interaction point, the hexapod base plate is visible.

Fig. 5 ▸ shows an overview of the imaging systems. To give free sight to the objectives, the catcher assembly has been removed. The hexapod is visible in the bottom, with the bellows leading to the pumping station. The X-ray beam enters from the right-hand side of the picture; on the left-hand side, the large detector flange is visible. The predominant part in the picture is the Z-shaped inline microscope with alignment stages. In the back, the side-view microscope manipulator with three different objectives is visible.

### 5.1. Inline microscope   

**Figure 6:**
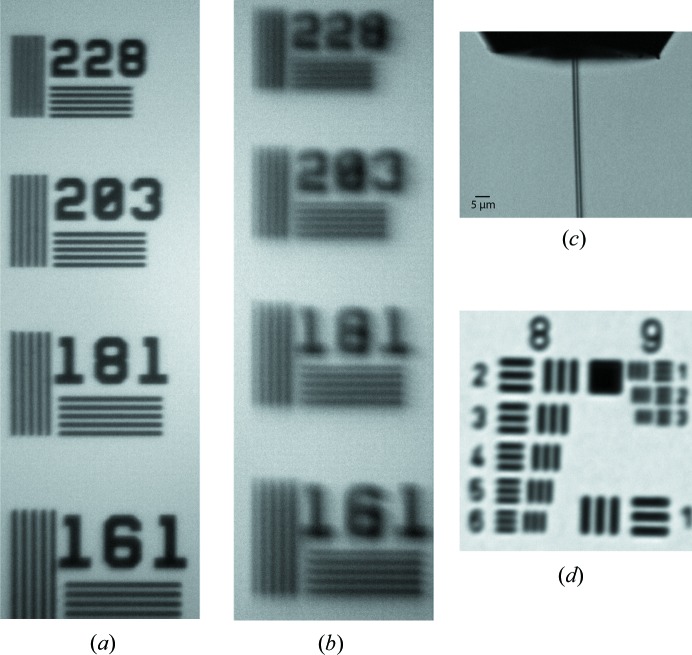
Example images from the inline and side-view microscopes. (*a*) A resolution standard imaged with an unmodified 10× objective in the inline microscope. The numbers next to the field of lines give the number of line-pairs per mm. A line in the 228 field is 2.2 µm thick. (*b*) The same resolution standard imaged with a centre-bored 10× objective. Additional scattering close to the centre bore produced blurring of the image and shadow images. 203 line pairs per mm can still be resolved. (*c*)–(*d*) Images from the side-view microscope. (*c*) Running liquid jet with a 5 µm scale bar. (*d*) 1951 USAF resolution test chart. Element 4 in group 8 gives 362 line pairs per mm.

The inline microscope is mainly constructed from commercially available optical components. This keeps the costs low and eases replacement and upgrades of the components. The microscope body consists of a 1 inch tubing system with two mirror cubes. The lower cube is equipped with a 45° mirror with a 2 mm-diameter centre bore to let the X-rays pass through and reflect the microscope optical axis 90° upwards into the microscope tube. The lower cube also carries the thread for mounting the centre-bored objective. Directly on top of this cube follows the mount for a 200 mm achromatic tube lens. The tube folds through the other cube, where an inline light source can be coupled with a 50/50 beamsplitter. The upper port of this beamsplitter cube allows coupling of the light source via an SMA fibre connector. The right-hand side extends the cube and ends with a c-mount camera port. Figs. 6 ▸(*a*) and 6(*b*) give an impression of the typical resolution of the inline microscope with 10× objective.

The inline microscope is mounted on a five-axis manipulation system controlled by two-phase stepper motors and absolute optical encoders. The two horizontal stages allow 25 mm travel, the vertical axis is 50 mm. Two 360° rotation stages allow pitch and yaw around the back hole of the cube where the X-rays enter the microscope.

The camera is operated at 10 Hz, triggered by the European XFEL bunch clock. It is a commercial camera for in-air use. In order to operate it in a vacuum, a copper braid has been connected to the electronics to conduct the excess heat into the chamber body.

### 5.2. Side-view microscope   

The side-view microscope is a split design with an objective changer under vacuum and in-air tube optics and camera. This is possible because of the free line-of-sight to the side of the chamber and is desirable because it allows mounting of any camera system onto the microscope. Available are 10 Hz commercial cameras synchronized with the European XFEL bunch trains as well as high-speed cameras for *in situ* studies of the liquid-jet stability.

In the vacuum, a three-axis linear manipulation system can move one of three infinity-corrected objectives into the optical path and align and focus it on the interaction point. A re-entrant flange with a standard viewport allows positioning of the camera with a tube lens outside the vacuum chamber to image through the objectives. Figs. 6 ▸(*c*) and 6(*d*) give an impression of the typical resolution of the side-view microscope with 10× objective.

## 6. Conclusion   

The described setup has been in operation at the SPB/SFX instrument since September 2017. Two of the first experiments measured data sets of lysozyme and jack bean proteins (Grünbein, 2018 ▸), and lysozyme and β-lactamase (Wiedorn, 2018 ▸), showing that MHz repetition rate SFX is feasible with the experimental setup. To adapt for different user needs and to improve sample delivery and nozzle exchange efficiency, slight modifications to the setup are applied for each experiment.
